# Supplementary material for: Time‑resolved multi-omic analysis of paclitaxel exposure in human iPSC‑derived sensory neurons unveils mechanisms of chemotherapy‑induced peripheral neuropathy
Source: Cell Death Dis. 2026 Feb 10;17(1):211. doi: 10.1038/s41419-026-08445-2 (PMC12921266; doi:10.1038/s41419-026-08445-2)
Supplement: Supplementary file 15 — Dataset 1. [file 41419_2026_8445_MOESM15_ESM.zip › CDD_Open_Data/04_RNA_Seq/Timeline_panel_plots_etc_2023-01-23_1609.zip!/Panel_Plots_etc/Evidence_Plots/6h/evidence_plot_standard contrasts_msigdb_reactome_6h_PTX_vs_DMSO_ID2_M29813_REACTOME_RESPONSE_OF_EIF2AK4_GCN2_TO_AMINO_ACID_DEFICIENCY.pdf]

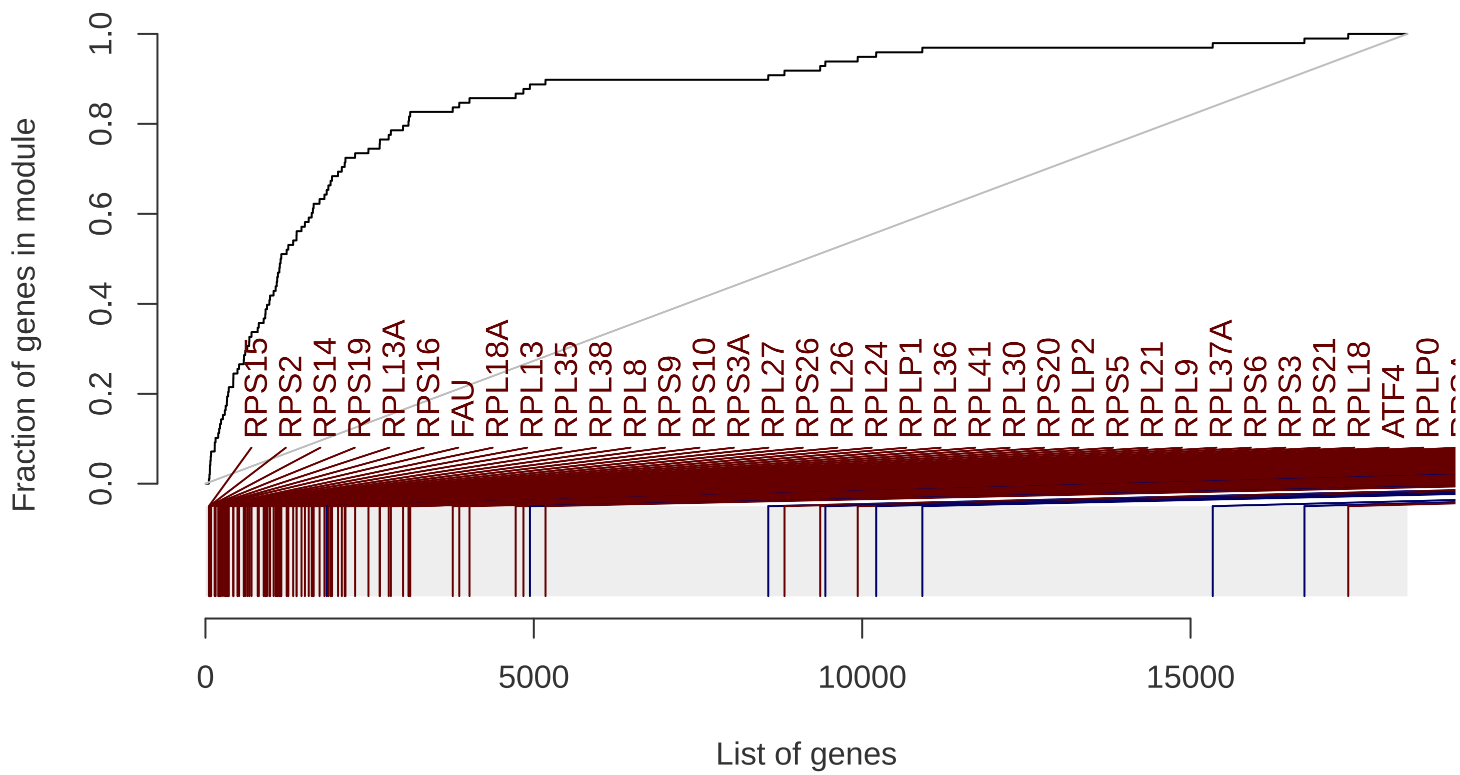
Fraction of genes in module
0.0
0.2
0.4
0.6
0.8
1.0
RPS15
RPS2
RPS14
RPS19
RPL13A
RPS16
FAU
RPL18A
RPL13
RPL35
RPL38
RPL8
RPS9
RPS10
RPS3A
RPL27
RPS26
RPL26
RPL24
RPLP1
RPL36
RPL41
RPL30
RPS20
RPLP2
RPS5
RPL21
RPL9
RPL37A
RPS6
RPS3
RPS21
RPL18
ATF4
RPLP0
0
5000
10000
15000
List of genes
